# Effects of family-based diabetes self-management education and support programme on support behaviour amongst adults with type 2 diabetes in Western Ethiopia

**DOI:** 10.1038/s41598-023-48049-w

**Published:** 2023-11-27

**Authors:** Dereje Chala Diriba, Doris Y. P. Leung, Lorna K. P. Suen

**Affiliations:** 1 School of Nursing, The Hong Kong Polytechnic University, Hong Kong, Hong Kong SAR China; 2 School of Nursing, Tung Wah College, Hong Kong, Hong Kong SAR China

**Keywords:** Diseases, Endocrinology

## Abstract

Support from family and peers may enhance the outcomes of diabetes management. This study reported the preliminary effect of a family-based diabetes self-management education and support (DSMES) programme on the perceived support status of people with diabetes and the family’s caregiver support behaviour amongst dyads living in Western Ethiopia. A 1:1 two-armed pilot randomised controlled trial (RCT) was conducted. A total of 76 dyads were recruited using the convenience sampling method and randomly assigned to either intervention or control groups. The control group continued the usual care, whereas the intervention group continued the usual care and additionally received a 12-h social cognitive theory (SCT)-guided, family-supported DSMES programme in the community. Generalised estimating equations models were computed to test the preliminary effects of the DSMES programme on the outcomes. P-value < 0.05 was set as statistically significant. The pilot RCT shows a statistically significant between-group difference in the changes in support needed at T1 (*d* = 0.88) and T2 (*d* = 1.35) and support received at T1 (*d* = 0.88) and T2 (*d* = 1.44). The DSMES programme has outperformed usual care with a medium effect size at T1 (*d* = 0.54) and a large effect size at T2 (*d* = 0.97) on the family’s supportive behaviour. Although the intervention group was not statistically significant at T1 (*d* = 0.43), a large effect size was obtained at T2 (*d* = 0.97) on the family’s non-supportive behaviour. A SCT-guided, family-supported DSMES programme produced a promising positive effect on enhancing the support needed and support received from their family/friends, and it also improved the family’s supportive behaviour. Thus, family support could be incorporated into DSMES programmes for diabetes management in Western Ethiopia. The trial was registered by the Chinese Clinical Trial Registry (http://www.chictr.org.cn); Registration number: ChiCTR2000040292.

## Introduction

Diabetes is a chronic disease that poses a significant burden on people with diabetes, families and healthcare systems^1^. In Africa, the burden of diabetes has increased due to multifaceted factors and problems. A few years back, sub-Saharan Africa (SSA) was challenged by communicable diseases. Currently, non-communicable diseases, including diabetes, have become the most challenging health problem in SSA^2^. Diabetes causes direct or indirect mortality amongst people with diabetes. If diabetes is left untreated, then it may lead to an overabundance of acute and chronic complications with varying physiologic functions, causing premature death^2^. According to the International Diabetes Federation^3^, approximately 6.7 million adults with diabetes die due to the disease. Ethiopia shares this diabetes-related mortality, and the number is increasing^4^.

Moreover, diabetes consumes the family caregiver’s time, especially since people with diabetes require care and emotional support and help in self-management^5^. Besides, the family may be involved in managing people with diabetes health and cover the drug costs when people with diabetes cannot afford management costs. Beyond the family, diabetes also imposes a considerable economic burden on the society with adverse effects^6^. The types of burden are comparatively higher amongst people receiving low social support^7^.

One of the challenges related to diabetes management in Ethiopia is family support. Family is important in social networking, in which the family members provide support for people with diabetes^8^. Family can demonstrate supportive or non-supportive behaviour or both to the patient’s management of diabetes. Supportive family behaviours include giving praise for following the diet, suggesting approaches that may help people with diabetes, helping to decide if changes occur to blood glucose, as well as encouraging them to participate in sports activities and eating and exercising together with people with diabetes. Supportive behaviour of the family may encourage the self-care behaviours of people with diabetes and discourage the negative ones^9–11^. By contrast, non-supportive family behaviours include nagging people with diabetes to perform self-care, criticising them for not exercising regularly, arguing about diabetes self-care activities and forcing them to eat foods that are not a part of a diabetic diet. Non-supportive family behaviour may lead to diabetes distress and negatively affect people with diabetes^12,13^. Thus, family caregivers need to understand their role in supporting their relatives with diabetes, particularly the supportive and non-supportive behaviours.

A previous study showed that family support is a predictor of self-care practise amongst people with diabetes^14^. It is a need to involve family caregivers according to a culture of the society and increasing their diabetes knowledge. Thus, the diabetes self-management education and support (DSMES) programme involving the culture of people is necessary. For example, eating pattern of people is affected in Ethiopian society due to tradition of consuming food with all family members from a communal plate^15^. Owing to this tradition, the chance is high for foods of family members to be mixed amongst those with and without diabetes. There is a limited studies reported the effectiveness of family based DSMES on perceived support status and family’s supportive behaviour. Educating the family can help to synergise dietary habits^16,17^ and diabetes self-management activities^18^.

We have conducted a randomised controlled trial (RCT) to examine the preliminary effectiveness of a family-supported, culture-based DSMES programme amongst people with type 2 diabetes (T2D) and their family caregiver in Western Ethiopia. In this article, we reported the results on perceived support status of people with T2D and support behaviours in family caregivers. Results on clinical outcomes in people with T2D were reported elsewhere^19^.

## Methods

This result is one part of the RCT, and a trial protocol was published elsewhere^20^.

### Trial setting

A hospital-based recruitment of samples was conducted between January and March 2021. The trial was conducted consecutively in a community of Nekemte, Western Ethiopia for 6 months.

### Trial design

A pilot randomised controlled trial with a 1:1 allocation was conducted.

### Samples

Adults with type T2D and one nominated family caregiver fulfilling the inclusion criteria were included in the trial. The inclusion criteria were—for people with T2D—people attending the hospital for monthly medical follow-up, taking antidiabetic agents and—for family caregivers—living with people with T2D and volunteer to provide support.

### Interventions

Dyads of people with T2D and one of the family caregivers were allocated to either the intervention group received the DSMES programme or continued usual care. Family-based intervention was selected because of the roles of family on encouraging, motivating and providing instrumental support when people with diabetes perform self-management behaviours such as preparing food, taking medication and performing physical activities. Previous literature pointed that the involvement of family members in the intervention as a good option to boost the control of glucose and monitor other clinical parameters for the cultural value of familyism^21,22^. Given the important role of the family in Ethiopian culture, particularly in preparing food for the whole family. One of the key innovations of the current intervention was highlighting the important role of family members in diabetes care, in particular, to provide support to family members to boost their supportive behaviours in the self-management care of people with diabetes in the cultural context.

The programme contains seven self-care behaviours for diabetes management (healthy diet, physical activity, medication, self-monitoring of blood glucose (SMBG), diabetes complications, healthy coping and problem-solving)^23^, diabetes-related misconceptions and foot care followed by home-based family support. The healthy diet and family support was developed focused on the culture of the society. The intervention protocol was designed based on the findings of the systematic review and meta-analysis on the effectiveness of self-management programme amongst adults with diabetes in Africa^24^ and constructs of the social cognitive theory^25^. Both people with T2D and their family caregivers were involved in the educational programme, and they set a goal to be performed when they returned home. The intervention sessions were supported with an education handbook, two videos and fliers. The review findings also indicate that face-to-face delivery and interventions for more than 10 h effectively improved most patient outcomes^24^. Hence, the intervention was given in face-to-face for 12 h, with six 2 h sessions on a weekly basis. An educational handbook includes (a) a brief introduction about diabetes and its management, including the definition, diagnostic criteria overview, risk factors, prevalence and management approaches, (b) a common diabetes-related myths and misconceptions in Ethiopia: The available myths and misconceptions, and (c) the seven content areas of self-care behaviours^23^ that include nutritional education for healthy diet addressing the general nutrition guidelines for diabetes, nutrition recommendation for Ethiopia and nutrient sources, including glycaemic index. In addition, the food estimation techniques, including the plate method, were included. Other self-care behaviours on recommendation of physical activity, medication, SMBG, healthy coping with psychosocial issues and problem-solving skills, diabetes complications and sick-day management, as well as foot care were addressed (Supplementary Table S1).

In addition, based on the social cognitive theory (SCT)—the theory which considers the learning occur in a social context, that is, in a dynamic and continuous interaction of the environment, the cognitive and person, and behaviour’ (reciprocal determinism—the intervention was delivered using the behavioural change strategies like weekly goal setting, discussions, demonstrations, plate method and rewards. The matching of behavioural change strategies with the SCT constructs was given in Supplementary Table S2. Goal setting is widely recommended in interventional studies related to diabetes self-management^26^, and it is even an effective strategy for ensuring behavioural change^27,28^. Thus, goal setting, experience sharing and group discussions were implemented in each session because learning occurs when the person develops and achieves a goal. Furthermore, goal setting encourages individuals to perform and achieve the goal in their daily life. The sharing of experiences by other individuals, focusing on their diabetes and taking part in group discussions on the raised points, enable the affected persons to learn. Moreover, verbal appraisal was implemented in each session, and appraisal feedback was shown to increase participation and change their behaviour. The plate method was implemented via a nutrition education session, demonstration in SMBG and medication and foot care video sessions. The SCT supports the pursuit of symbolising enhancing and sustaining behaviour. Healthy coping and problem-solving skills were also implemented. The coping mechanisms and problem-solving skills may reduce the anticipated risks of the behaviour. The plate method, demonstration and videos were linked to the symbolising capability and observation learning construct. The experience sharing and group discussion were related to vicarious capacity and observational learning construct. Motivators, such as verbal persuasion and rewards for best performers, were linked to the self-regulatory ability and reinforcement construct. Goal setting, healthy coping and problem-solving skills were related to forethought capacity and behavioural capability. The family support technique was implemented in each session, and it was related to self-efficacy and reciprocal determinism. While the discussions and goal setting were implemented weekly during intervention, demonstrations were performed on medication administration and plate method was used on nutrition education sessions. However, the reward was given at the end of the intervention for those performed best from each intervention group. The novelty of the intervention was (1) the inclusion of culturally specific nutritional knowledge and diabetes-specific misconceptions and myths, (2) the application of diversified behavioural change strategies and (3) the co-engagement of dyads in the intervention. The control group continued usual care provided by the hospital, which was pharmacological management approaches to diabetes.

### Outcomes

Perceived support status (support needed, support received and support attitudes) from people with T2D and diabetes family support behaviour from the family caregiver were assessed at baseline (T0) at recruitment, post-intervention (T1) immediately after intervention completed and follow-up (T2) at 2 months after T1.

### Outcomes measure

- People with T2D’s perceived support status (subscales: support needed, support received and support attitude): The diabetes care profile (DCP) support scale developed by the Michigan Diabetes Research Centre to measure the social and psychosocial factors of diabetes and its treatment^29^ was used to measure perceived support status of people with T2D. The scale has 18 items, with six items in each of the three subscales. One sample item in the support needed subscale is ‘I want plenty of help and support from my family or friends in following my meal plan’. The support received subscale’s sample item is ‘My family or friends help and support me a lot to follow my meal plan’. The support attitudes subscale includes a sample item: ‘My family or friends accept me and my diabetes’. Internal consistency of the DCP support scale was showed to be acceptable to excellent, with Cronbach’s alpha values ranged from 0.73 to 0.93^30,31^. The mean score of the subscales was calculated, with higher scores indicating stronger perception to obtain support from families or peers.
  The psychometric testing of DCP support–Afaan Oromoo scale results reveals an acceptable reliability with Cronbach’s alpha of 0.75. Whilst the support needed scale showed good reliability (Cronbach’s alpha = 0.81), the support received (Cronbach’s alpha = 0.75) and support attitudes (Cronbach’s alpha = 0.71) scales showed acceptable reliability^32^. In the current study, Cronbach alpha of the Afaan Oromoo version of the DCP support scale at baseline was 0.886, with 0.817 for support needed, 0.861 for the support received and 0.737 for support attitude subscales.
- The family caregiver’s supportive behaviour: Family behaviour is a pattern of behaviour that a family uses for dealing with family situations^33^, and was measured using the diabetes family behaviour checklist (DFBC)—Afaan Oromoo scale in this study. The original version of the DFBC scale consists of nine items in supportive and seven items in non-supportive subscales^34^. The DFBC showed an acceptable internal consistency between 0.71 and 0.86^34–36^, and it was used to measure family behaviour in previous studies^37,38^. The translation, cultural adaptation and psychometric testing of the scale were conducted, which resulted to some modifications to the original scale. A two-way mixed model with mean ratings at 95% CI intraclass correlation coefficient (ICC) was computed to assess the agreement of the DFBC at a 1-month interval^32^. The ICC of the overall DFBC was 0.593, which shows moderate consistency^39^. The ICC of the non-supportive DFBC subscale was 0.725, which shows moderate, whereas the supportive DFBC's ICC was 0.761, which reveals good test–retest reliability. The DFBC–Afaan Oromoo scale contains 12 items (six in supportive and six in non-supportive subscales). Each item is rated with a five-point Likert scale ranging from 1 (never) to 5 (at least once a day). The mean score was computed to assess the pooled family behaviour and higher scores indicated stronger family perception to support people with diabetes^40^. In this pilot RCT study, Cronbach alpha of the Afaan Oromoo version of the DFBC scale at baseline was 0.839, with 0.705 for supportive and 0.701 for non-supportive subscales.

### Sample size

Seventy-six dyads (people with T2D and family caregiver) were involved in the trial. The recommendation of Hertzog^41^ for pilot RCT was assumed, with 20% attrition. They equally divided either to the intervention group or control group.

### Participant recruitment

Participant recruitment was done using a convenience sampling technique for people with T2D, whereas nomination of family caregiver was done by people with T2D.

### Randomisation and masking of participants

After participant recruitment, randomisation was done by using a free online software (https://www.randomizer.org/). After random codes were generated, the codes placed in a serially numbered opaque and sealed envelopes. After baseline data collected, data collectors opened the sealed envelope and informed the group assignment’s information of those assigned to the intervention group to intervention facilitators. Blinding of the participants and intervention providers was not maintained due to intervention nature; however, the outcome assessors and data analyst were masked through coding to the group allocation. We also asked the participants to keep the intervention received confidential and not to be shared with others.

### Data collection procedure

Data was collected using face-to-face interview technique at all time points.

### Data analysis

Generalised estimating equations (GEEs) models were computed to estimate the between-group differences in the effectiveness of the DSMES programme intervention on the outcomes over the study period. The effect sizes of the DSMES programme on the outcomes were estimated using between-group Cohen's d. The value of Cohen's d of 0.2, 0.5 and 0.8 indicate small, medium and large effect sizes, respectively^42^. Intention to treat principle was applied and *p*-value < 0.05 considered as a statistically significant.

### Ethical approval

Ethical approval for the study was obtained from the Human Subjects Research Ethics subcommittee of The Hong Kong Polytechnic University (Reference number: HSEARS20201019003). Permission to recruit participants and collect data was obtained from the Nekemte Specialised Hospital medical director, and consent to gather the intervention group in the community was obtained from two selected *Kebeles*’ chairpersons. The study follows the Declarations of Helsinki^43^. Informed consent was obtained from all the study participants.

## Results

### Feasibility of the study

**Figure 1:**
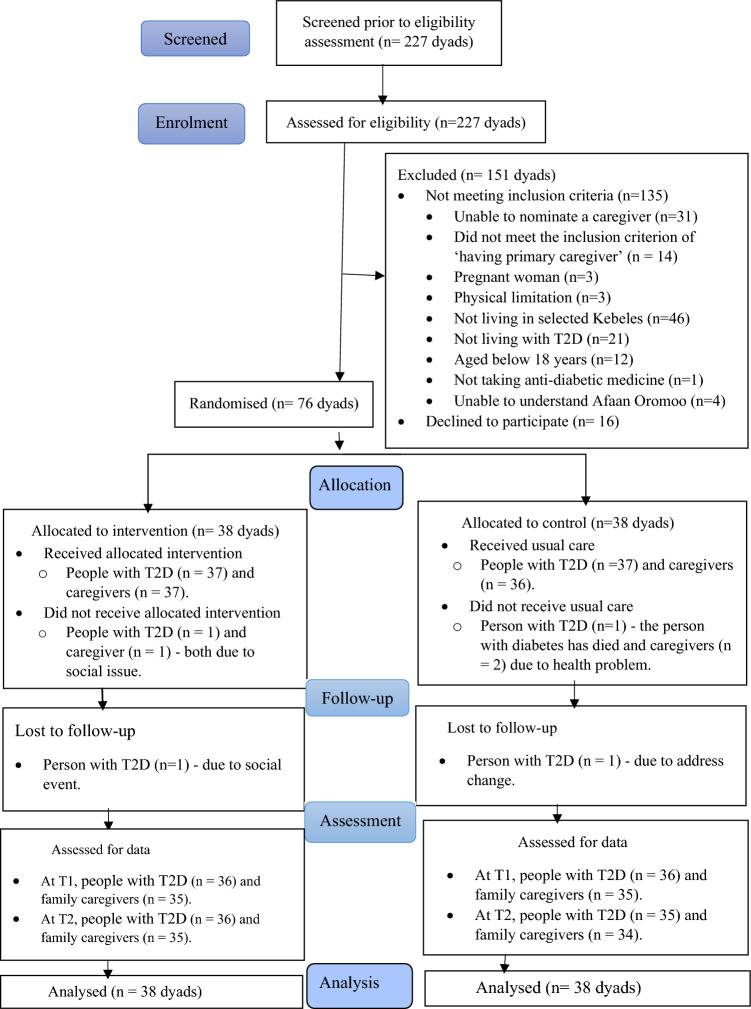
The CONSORT flow chart of the study procedures.

A total of 76 dyads were participated in the study. Out of the 38 dyads allocated to the intervention group, 37 people with diabetes (97.4%) and 37 family caregivers (97.4%) completed the intervention sessions due to a social event. Low attrition rates were obtained for people with diabetes, and slightly higher attrition rates were observed for family caregivers. However, there was no statistically significant between-group differences in people with diabetes and family caregiver’s attrition rate (Fig. 1).

### Demographic characteristics

The mean age was 49.4 ± 10.2 years for people with diabetes and 36.0 ± 12.2 years for family caregivers. More than half (55.3%) of people with diabetes and more than two-thirds (69.7%) family caregivers were females. Nearly two-thirds of people with diabetes (64.5%) received support from their spouse, with a larger proportion (71.1%) in the control group than in the intervention group (57.9%). Nearly a third of the family caregivers (31.6%) had attended secondary school and 30.3% were employees. There was no statistically significant between-group differences were obtained amongst dyads sociodemographic characteristics and support behaviours at baseline.

### Interventional effects

#### Perceived support status

The GEE results show a statistically significant difference in the changes in support needed and support received over the study period between the two groups, with the DSMES programme has outperformed usual care with large effect sizes both at T1 and T2. However, no statistically significant difference in the changes in support attitudes over the study period between the two groups, and the usual care has outweighed the DSMES programme approach with a small effect size both at T1 and T2 (Table 1).

**Table 1:** GEE results of support status of people with diabetes by the group over the study period.

Time	Intervention group (n = 38)	Control group (n = 38)	Group * time interaction effect				Cohen’s d
	Estimated mean (SE)	Estimated mean (SE)	β	95% CI		p-value	
Support needed							
T0	4.04 (0.17)	4.44 (0.14)					
T1	4.31 (0.16)	3.55 (0.16)	1.175	0.591	1.760	< 0.001	0.88
T2	4.68 (0.10)	3.46 (0.17)	1.636	1.106	2.166	< 0.001	1.35
Support received							
T0	4.17 (0.17)	4.37 (0.15)					
T1	4.30 (0.16)	3.37 (0.17)	1.123	0.558	1.688	< 0.001	0.88
T2	4.81 (0.07)	3.26 (0.19)	1.750	1.210	2.290	< 0.001	1.44
Support attitudes							
T0	4.78 (0.07)	4.75 (0.06)					
T1	3.21 (0.10)	3.13 (0.12)	0.061	− 0.293	0.416	0.735	0.06
T2	3.11 (0.07)	3.05 (0.09)	0.044	− 0.268	0.356	0.783	0.07

#### Family caregiver’s supportive behaviour

The GEE result shows a statistically significant difference in family caregiver’s supportive behavioural changes over the study period between the two groups. The DSMES programme has outperformed usual care with a medium effect size at T1 (*β* = 2.743, *p* = 0.020, *d* = 0.54) and a large effect size at T2 (*β* = 7.087, *p* < 0.001, *d* = 0.97) on supportive behaviour. A statistically significant difference in the changes in family caregiver’s non-supportive behaviour at T2 (*β* = 6.361, *p* < 0.001, *d* = 0.91) but not statistically significant at T1 (*β* = 2.342, *p* = 0.056, *d* = 0.43) between the two groups, with the usual care has outweighed DSMES programme at T1(Table 2).

**Table 2:** GEE results of supportive behaviour of family caregivers by the group over the study period.

Time	Intervention group (n = 38)	Control group (n = 38)	Group * time interaction effect				Cohen’s d
	Estimated mean (SE)	Estimated mean (SE)	β	95% CI		p-value	
Supportive behaviour							
T0	17.65 (1.02)	15.55 (0.68)					
T1	22.55 (0.79)	17.70 (0.61)	2.743	0.435	5.050	0.020	0.54
T2	25.16 (0.52)	15.97 (0.85)	7.087	3.861	10.313	< 0.001	0.97
Non-supportive behaviour							
T0	16.89 (0.99)	14.74 (0.55)					
T1	19.58 (0.71)	15.08 (0.61)	2.342	− 0.060	4.744	0.056	0.43
T2	23.31 (0.53)	14.79 (0.82)	6.361	3.263	9.460	< 0.001	0.91

## Discussion

The study found that the DSMES programme intervention had favourable effects on improving the support needed and the support received, with medium to large effect sizes amongst people with diabetes in Western Ethiopia. Support needed and support received were increased but eventually decreased with respect to support attitudes in the intervention group compared with those in the control group. Similarly, the family caregiver’s supportive behaviour was enhanced over the study periods, but more non-supportive behaviours were observed only during the follow-up.

The preliminary DSMES programme significantly improved the support needed and received outcomes compared with the usual care. The main possible reason for better support needed and received from friends or families was the involvement of family caregivers in the intervention. According to SCT, family support is an environmental factor that influences personal behaviours. The support seeking behaviour of people with diabetes may be increased because the family caregivers co-attended the intervention and set a goal together, which may give them intimacy about diabetes and its management. A finding from a systematic review that included 23 studies to see the effect of family support on diabetes outcomes amongst people with T2D showed a positive outcome on perceived support^44^. In this study, nearly two-thirds (64.5%) of people with diabetes received support from spouses. The support needed from their friend or family may be enhanced because of frequent interaction between their spouse and people with diabetes. Most spouses live in the same home in Ethiopia and decide on most issues together. Hence, the chance to seek and receive support from their spouse is more likely compared with friends and other family members. More than two-thirds (69.7%) of family caregivers were females. In Ethiopia, females provide home-based support for a sick and diseased person; in contrast, males are responsible for most outdoor activities. Females provide better informal care for people with chronic illnesses^45^. Females are more likely to discuss and support purchasing and preparing foods, delivering drugs, foot washing equipment, assisting in blood glucose testing, and supporting on sick days^46^.

The families obtained a healthy diet for a person with diabetes and the technique of food estimation. Their diabetes knowledge might have been increased by observation and experience sharing on diabetes self-management (DSM). A previous study reported that DSM education improved social support status^47^. These findings are consistent with the result of this study, perhaps due to family involvement because their involvement will promote healthy behaviour and well-being^48^. Hence, it can be recommended that family involvement in the intervention, increased interaction between people with diabetes and family or friends and awareness creation for people with diabetes on the importance of social support in DSM is needed.

In contrast, the DSMES programme has not effectively improved the support attitudes in the intervention group compared with the usual care. Verbal persuasion and increased behavioural capacity were designed to boost the support attitudes. A community-based study implementing DSM education intervention found that intervention was ineffective compared with usual care^49^. The study reported that empowerment was ineffective in increasing social support, implying support attitudes may not be related to verbal persuasion, behavioural capacity, and empowerment, indicating that support attitudes may take intensive intervention and lower intervention duration.

The DSMES programme produced positive and significant results in terms of the family caregiver’s supportive behaviour over the study period between the two groups. This finding is similar with mobile app delivered family supported self-management intervention^50^. This positive change in the family caregiver’s supportive behaviours could be related to the DSMES programme providing a specific component to clarify the role of the family caregivers in diabetes management, further encouraging family caregivers to provide continuous support to their relatives with diabetes. The supportive behaviour of the family may encourage the self-care behaviours of the person with diabetes and discourage the negative ones^9–11^. The family members obtained education on diabetes, its management, their roles in DSM and the misconceptions about diabetes in Ethiopia. Although most previous studies supporting family involvement in the intervention produced positive outcomes, some researchers argued that the roles of the family should be clearly stated in the intervention^51^. This study clearly defined the family’s roles in each session. Defining the family roles might be the reason for the effective supportive behaviours in family caregivers in the intervention group in this study.

The RCT conducted in Jimma University Medical Centre, Ethiopia, reported that DSM education was successful in increasing diabetes knowledge^52^. Diabetes-related knowledge may be one reason affecting the supportive behaviour of family members. Education on diabetes and self-management was given, as these elements could change their knowledge and behaviour to support relatives with diabetes. Weekly goal setting was set to provide support, further increasing their supportive behaviour. Another possible reason for the enhanced family caregiver’s supportive behaviour may be the increased self-efficacy owing to the intervention. Local diabetes-related misconceptions and myths were also addressed in the intervention. Group discussions and experience sharing on family support from participants were raised in the intervention sessions. Observational learning could help them develop the positive supportive behaviour of families who attended the intervention. Hence, a good recommendation is to include education on local diabetes misconceptions and myths, sharing positive experiences, clearly defining the roles of family in DSM, and arranging discussions on unclear issues.

On the other hand, the family caregivers in the intervention group held non-supportive behaviours at T1 than the usual care. This is also consistent with previous study^50^. Presumably, the family members might have been overwhelmed by some diabetes management-related behaviours, such as the non-compliance to DSM of persons with diabetes, leading them to feel annoyed and manifest non-supportive behaviours. Another possible reason for the family’s non-supportive behaviour might be related to the items in the scale. For example, nagging a person with diabetes about blood-glucose testing and not following dietary recommendations may not be a non-supportive behaviour in Ethiopia. In addition, the items about criticising the person with diabetes for not recording the results of blood tests, arguing about diabetes self-care activities and eating foods that are not part of their diabetic diet are not considered non-supportive behaviours amongst the Ethiopian population. Nevertheless, further support to family caregivers in the intervention and the corresponding time requirement may be considered, such as seeking the help of healthcare workers in the community to help with the awareness creation about DSM activities. In this study, only 11.8% of the participants received insulin and metformin as prescribed in BID (twice a day), usually taking them in the morning and at night. Thus, families may not consider the person’s sleeping time because they usually sleep after taking a drug; hence, it may not be regarded as a non-supportive behaviour. Encouraging family supportive behaviour is necessary to positively affect most diabetes outcomes.

Using validated diabetes specific scales to measure the outcomes is one of the strengths of the study. As the study was a pilot study, it can only provide a preliminary result, which needs a full scale RCT to ensure the effects of DSMES programme on the support behaviour of the dyads. Translation and psychometric testing of the scales were done on the target population which resulted in some modifications in the DFBC scale, further testing of the modified Afaan-Oromoo version of the DFBC scale are needed to examine the cultural adaptation in the Ethiopian population. In addition, the study did not address the qualitative study to see the intervention acceptability and there was a potential for information contamination as the pilot was conducted in one hospital and nearby community.

## Conclusions

A SCT-guided, family-supported DSMES programme produced a promising positive effect on enhancing the perceived support from their family/friends, and it also improved the family’s supportive behaviour. Thus, family support could be incorporated into DSMES programmes.

## Implications of the study

The pilot RCT findings showed that the DSMES programme positively affected the perceived social supportive behaviour of people with diabetes and the family caregiver’s supportive behaviour. The preliminary results, effect sizes, feasibility and acceptability of the intervention were identified. Future research is warranted to evaluate the DSMES programme’s effectiveness on a large sample size. The developed DSMES programme (educational handbook, videos and flyers) can be used in diabetes education by healthcare institutions for people with T2D–family caregiver dyads. Hospital-based diabetes education using this programme can be conducted during the morning session, and it offers an option for establishing community-based diabetes education centres and delivering outreach diabetes education.

### Supplementary Information

Supplementary Tables.

## Data Availability

The dataset used and analysed during the current trial is available from the first author on rational request.
