# Supplementary material for: Effects of family-based diabetes self-management education and support programme on support behaviour amongst adults with type 2 diabetes in Western Ethiopia
Source: Sci Rep. 2023 Nov 27;13:20867. doi: 10.1038/s41598-023-48049-w (PMC10682375; doi:10.1038/s41598-023-48049-w)
Supplement: Supplementary file 1 — Supplementary Tables. [file 41598_2023_48049_MOESM1_ESM.docx]

**Effects of family-based diabetes self-management education and support programme on support behaviour amongst adults with type 2 diabetes in Western Ethiopia**

**Dereje Chala Diriba^1^, Doris Y.P. Leung^1^, Lorna K.P. Suen^2*^**

**^1^School of Nursing, The Hong Kong Polytechnic University, Hong Kong**

**^2^School of Nursing,** Tung Wah College, Hong Kong

***Correspondence**

Email: [lornasuen@twc.edu.hk](mailto:lornasuen@twc.edu.hk)

Supplementary Table S1. DSMES programme topics, contents and activities and delivery schedule for dyads

| **Week and session** | **Topic** | **Content and activities** |
| --- | --- | --- |
| 1 | Brief introduction about diabetes mellitus  Diabetes-related misconceptions in Ethiopia | **The joint session for people with diabetes and family caregivers**   - Interactive lecture   - Definition, diagnosis criteria, classification, pathophysiology, and clinical features of diabetes   - Risk factors of T2D   - Prevalence of diabetes in Ethiopia   - Management of diabetes   Activity: Group discussion on symptoms of diabetes and diagnostic criteria   - Interactive lecture   - State common misconceptions related to diabetes   - Facts about misconceptions - Activities   - Experience sharing about the misconceptions held by people with diabetes and family members   - Group discussion on how to reduce or avoid such misconceptions - Goal setting   - Set goals and strategies for increasing awareness about misconceptions   **Role of the family caregiver**   - Hold an informal discussion with another family member on awareness creation on diabetes clinical features and misconceptions (information support) |
| 2 | Nutrition education | **The joint session for people with diabetes and family caregivers**   - Interactive lecture   - Goals of nutrition therapy   - General nutrition guidelines for diabetes   - Nutrition recommendations for Ethiopia (focusing on the western part)   - Nutrient sources of foods (the foods that raise sugar levels and those that do not) - Activities   - Experience sharing of dietary habits and meal planning   - Group discussion on the food sources of sugar - Goal setting   - Set meal plan for the coming week   **Roles of the family caregiver**   - Support people with diabetes in the selection of healthy food - Purchase a healthy diet - Cook the food |
| 3 | Nutrition education (continued) | **The joint session for people with diabetes and family caregivers**   - Interactive lecture   - Estimating food portions (using plate model)   - Traditional food and drink intake (focusing on the Western Ethiopian culture)   - Recommended drinks   - Food planning tips before travelling   - Meals during periods of fasting (Christian and Muslim fasting) *fasting in the Ethiopian context means going without meat or eggs or butter for Orthodox Christians (i.e. vegetarian +/- fish) rather than not eating. This is on certain days of the week and periods of the year, for example before Easter, and for Muslims, they do not eat for a half day, on the daytime like in Ramadan). - Activities   - Experience sharing in estimating food portions   - Group discussion on food intake whilst fasting - Goal setting   - Meal planning and portion estimation for the coming week   **Roles of the family caregiver**   - Support people with diabetes by estimating healthy meal portion - Discuss with other family members a healthy portion of food - Promote people with diabetes to take a healthy portion of drinks, including alcohol |
| 4 | Physical activity and medication | **The joint session for people with diabetes and family caregivers**   - Interactive lecture   - Benefits of physical activity   - Recommendations of physical activity for diabetes   - Cautions on physical activity   - How to use local facilities for physical activity - Activity   - Experience sharing concerning the physical activity - Goal setting   - Set goal to perform physical activity - Interactive lecture   - Medication   - Intake of oral hypoglycaemic agents   - Insulin injection techniques (video watch)   - Benefits of medication adherence - Activity   - Experience sharing on self-medication practise and strategies for medication adherence - Goal setting   - Set goals to increase medication adherence and self-injection   **Roles of family caregivers**   - Encourage people with diabetes to perform physical activity for 30 minutes/day for at least five days/week - Walk with people with diabetes for at least 30 minutes two days/a week - Remind people with diabetes to receive medication |
| 5 | SMBG and foot care | **The joint session for people with diabetes and family caregivers**   - Interactive lecture   - Benefits of SMBG   - When to check blood glucose   - How SMBG improves diabetes outcomes   - Benefits of foot care   - Risk factors for foot ulcer and amputation in diabetes   - What and when to perform foot care   - Personal care in foot care (Video watch) - Activity   - Experience sharing on SMBG and foot care - Goal setting   - Set goals to monitor blood glucose and perform foot care for one week   **Roles of family caregivers**   - Assist blood glucose testing - Help people with diabetes decide if the change has been made on the blood level. - Encourage people with diabetes to record their blood test results - Support people with diabetes in choosing shoes - Assist in performing personal foot care |
| 6 | Coping with psychosocial issues and problem-solving skills  Diabetes complications and sick-day management | **The joint session for people with diabetes and family caregivers**   - Interactive lecture   - Sources of psychosocial issues and their features   - Effective coping strategies   - Problem-solving skills - Activity   - Experience sharing on psychosocial issues encountered and coping strategies used   - Group discussion on steps of problem-solving skills   - Testimonies from the success story - Goal setting   - Take one coping strategy and set an action plan - Overview of diabetes complications   - Acute and chronic complications   - Why complications develop   - Sick-day management - Activities   - Experience sharing on the sick-day management   - Group discussion on strategies to reduce complications - Goal setting   - Set a goal to prevent complications and take action if complications develop   **Roles of family caregivers**   - Provide emotional support - Assist in the implementation of relaxation techniques - Establish survival skills and develop an action plan with all family members. - Congratulate people with diabetes for sticking to diabetes self-care activities - Establish companionship with people with diabetes - Assist in the sick-day management - Encourage to seek medical care if a complication happens   Conclusion of all sessions |

Supplementary Table S2. Behavioural change strategies and SCT constructs addressed in the DSMES programme

| **Behavioural change techniques** | **SCT construct addressed** | **Learning activities** |
| --- | --- | --- |
| Goal setting and developing an action plan | Self-efficacy | - Participants shared their experiences of performing self-care behaviours in all sessions - Set the goal and action plan to achieve the goal - The goal was set by dyads together and was facilitated by intervention facilitators - The action plan was developed for a week and implemented in the corresponding week |
| Verbal persuasion and awarding glucometer | Reinforcement | - The verbal appraisal was given after the dyads presented their achievements at the recap session - Awarded to best performers to sustain their behaviour |
| Using the plate model | Observational learning | - A plate model was used to visualise the meal portions |
| Group discussion and experience sharing | Observational learning | - Group discussion and experience sharing on managing diabetes were discussed in all sessions - Participants were asked about the lessons they had learnt |
| Healthy coping and problem-solving skills | Behavioural capability | - Healthy coping and problem-solving strategies to overcome diabetes-related challenges were delivered - Testimonies from the success stories were shared |
| Provide family support | Self-efficacy  Reciprocal determinism | - The family was involved in all sessions of the DSMES - The family provided home-based support - The roles of the family were communicated and evaluated by educators |
| Video display and demonstration of skills to encourage mastery in learning | Observational learning | - An educational video was displayed at the end of every session to demonstrate the desired skills, such as foot care and self-injection of insulin - Demonstration of meal estimation (portion estimation) |
